# Supplementary material for: Pre-diagnostic trajectories of lymphocytosis predict time to treatment and death in patients with chronic lymphocytic leukemia
Source: Commun Med (Lond). 2022 May 12;2:50. doi: 10.1038/s43856-022-00117-4 (PMC9098503; doi:10.1038/s43856-022-00117-4)
Supplement: Supplementary file 1 — Supplementary Information [file 43856_2022_117_MOESM1_ESM.pdf]

Supplementary Information

Title: Pre-diagnostic trajectories of lymphocytosis predict time to treatment and death in patients with chronic lymphocytic leukemia

Authors: Michael Asger Andersen[1,2], Mia Klinten Grand[3], Christian Brieghel[1], Volkert Siersma[3], Christen Lykkegaard Andersen[1,3] and Carsten Utoft Niemann[1,4]

[1] Department of Hematology, Rigshospitalet, Copenhagen University Hospital, Copenhagen, Denmark

[2] Department of Clinical Pharmacology, Bispebjerg Hospital, Copenhagen, Denmark

[3] The Research Unit for General Practice and Section of General Practice, Department of Public Health, University of Copenhagen, Copenhagen, Denmark

[4] Institute for Clinical Medicine, Copenhagen University, Copenhagen, Denmark

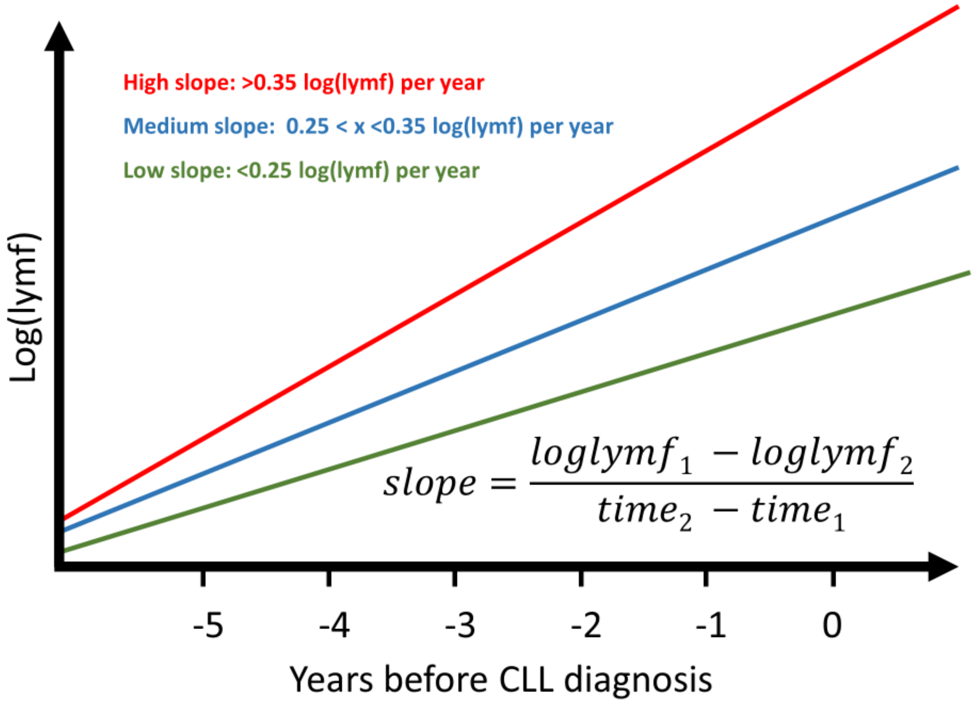

Supplementary Figure S1 *Calculating the slope group before diagnosis.*

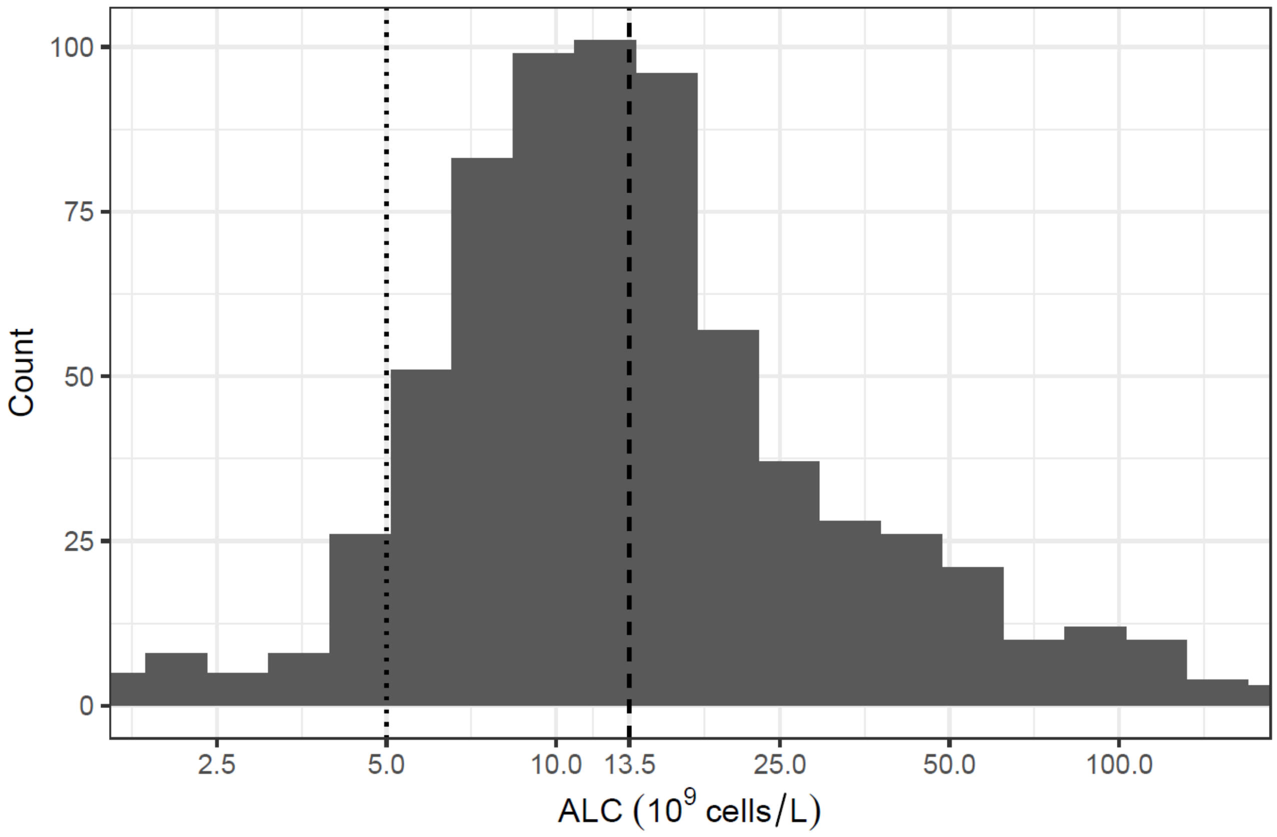

Supplementary Figure S2 *Histogram of ALC at time of diagnosis for all patients included in the study.*

The dotted line indicates the threshold of 5 (10^9 cells/L) and the dashed line indicates the median of the observed ALC at the time of diagnosis.

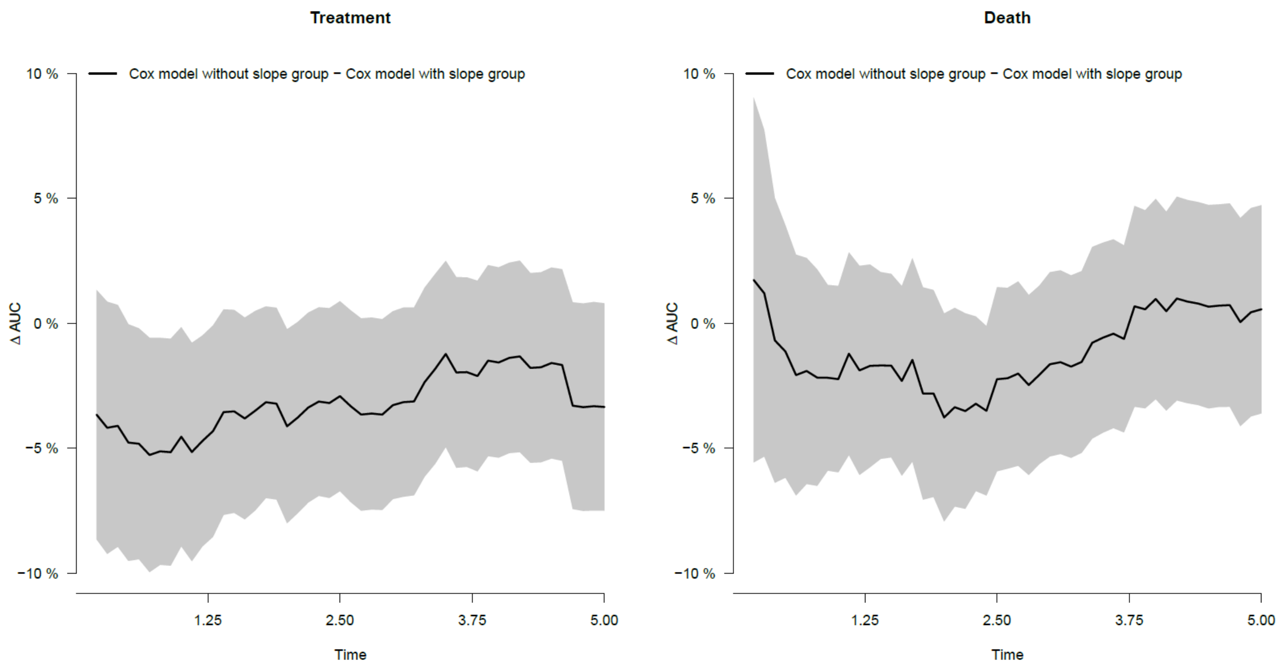

Supplementary Figure S3 Difference in AUC over time for treatment (left graph) and death (right graph) between the Cox model where the slope group was not included (Cox models without slope group) and the Cox model where the slope group was included (Cox models with slope group). A negative difference in AUC, thereby, means a smaller AUC at a given timepoint for the Cox models without slope group compared to the Cox models with slope group. For treatment the addition of slope groups improved the AUC at year 1 with 4.5 percentages points and 4.1 percentages points at year 2. For death AUC was improved with 2.2 percentage points at year 1 and 3.8 percentage points at year 2. The shaded areas are the 95 % confidence interval.

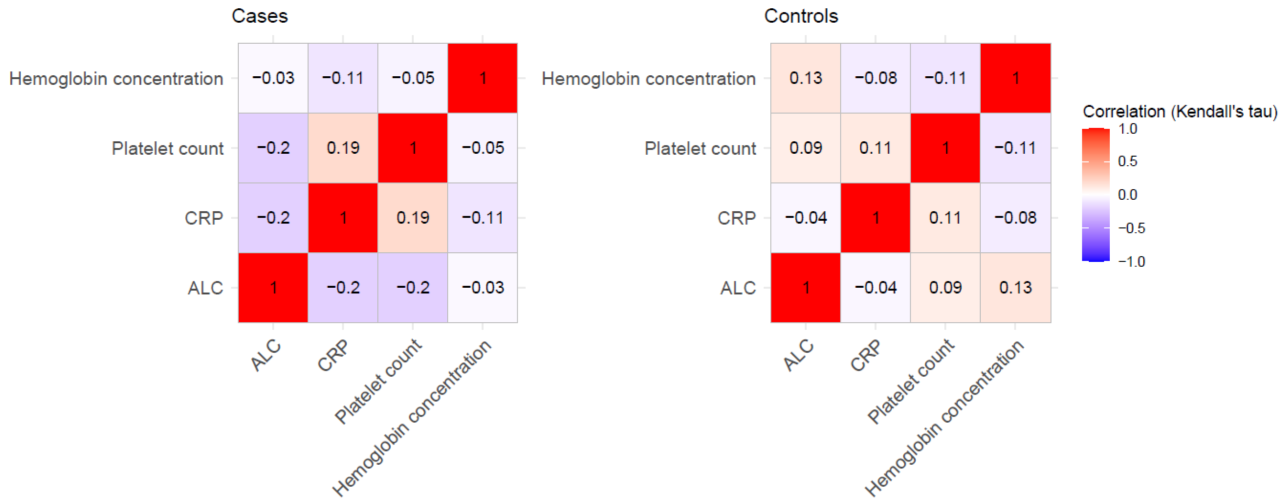

Supplementary Figure S4 Correlation between ALC, CRP, platelet count and haemoglobin concentration prior to diagnosis. Correlations were calculated based on a randomly selected observation for each person for cases and controls. Due to the non-linear relationship between some of the biomarkers Kendall's tau was used.

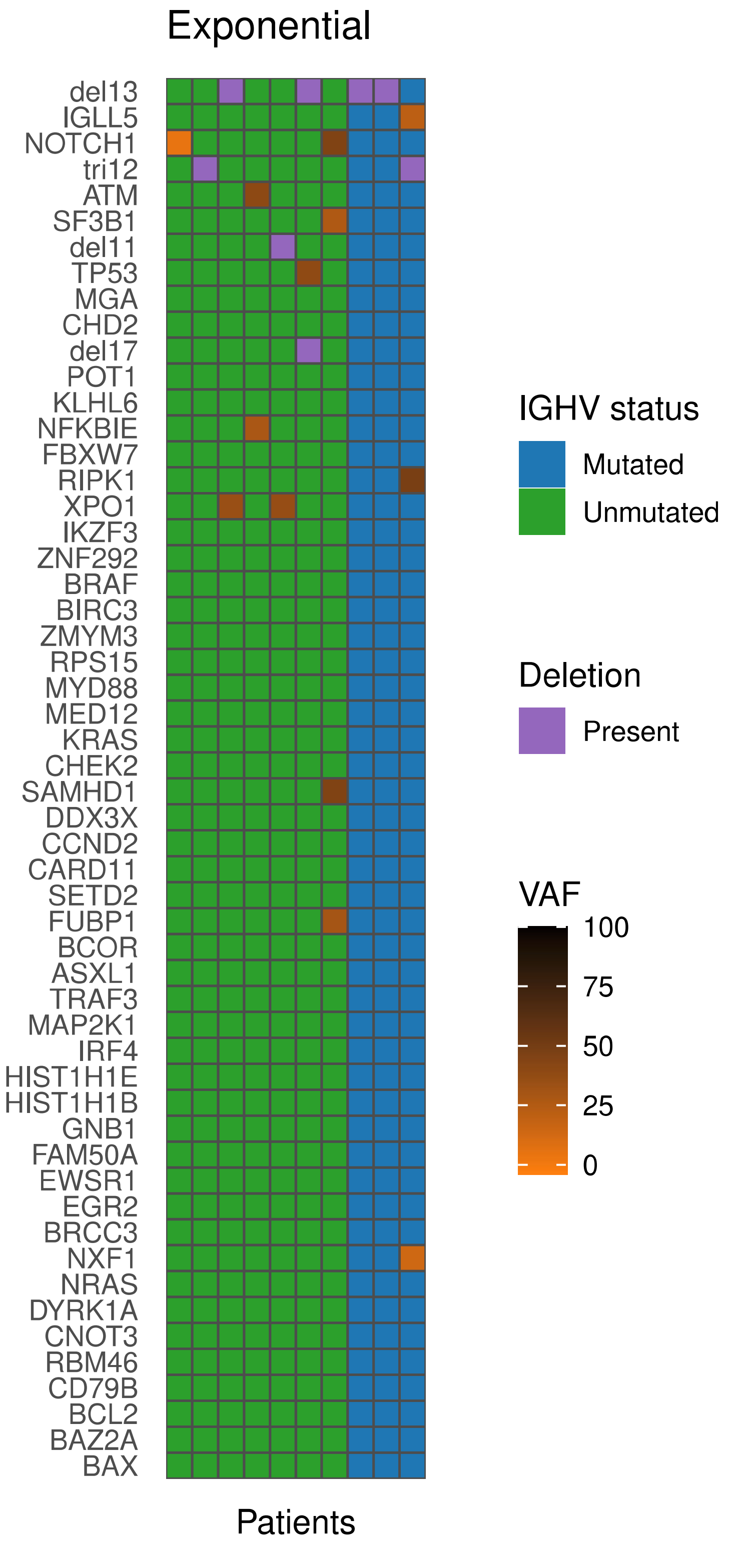

Supplementary Figure S5 *Growth dynamics and genetic changes in naturally progressing CLL.*

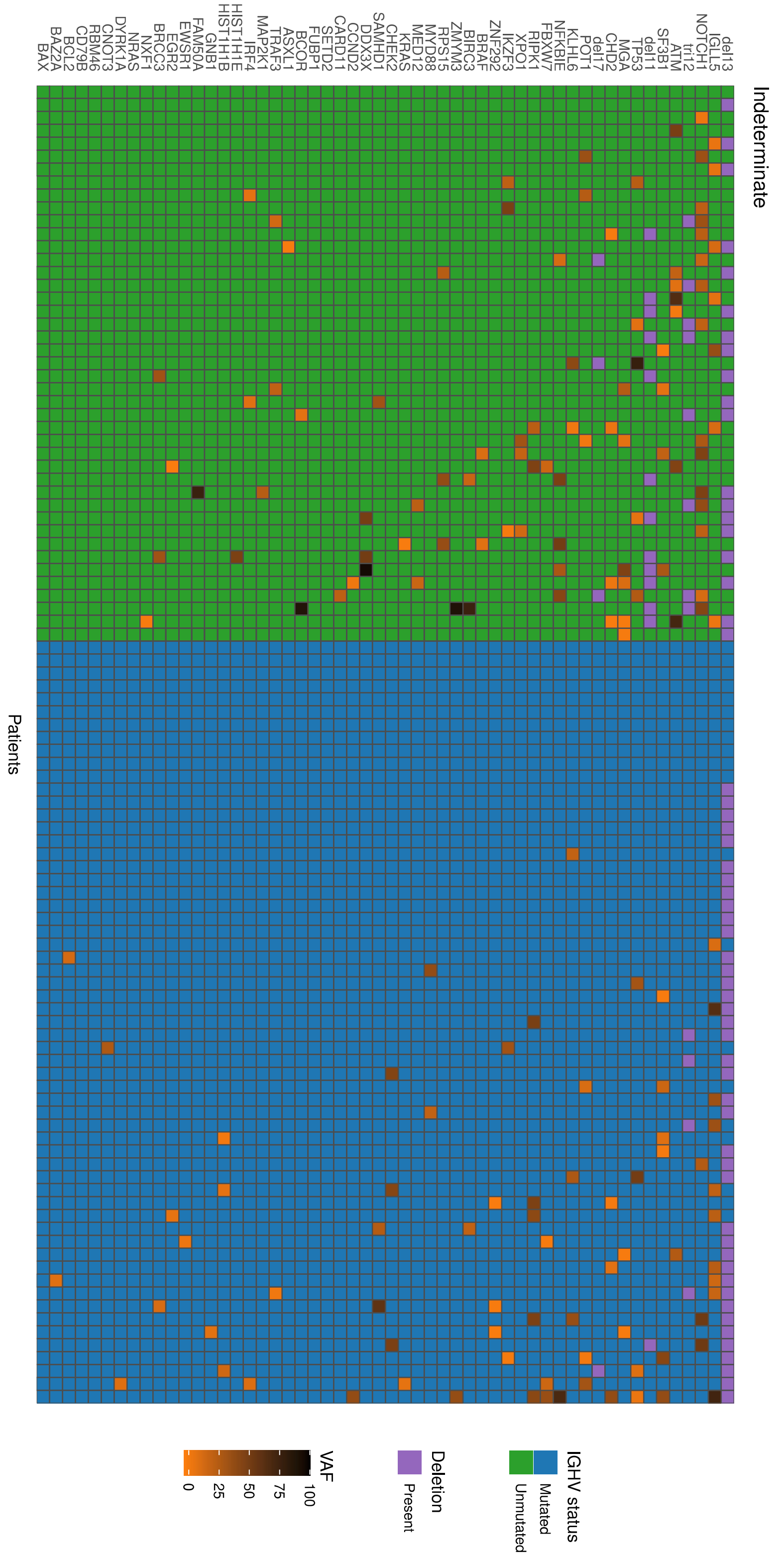

Supplementary Figure S6 *Growth dynamics and genetic changes in naturally progressing CLL.*

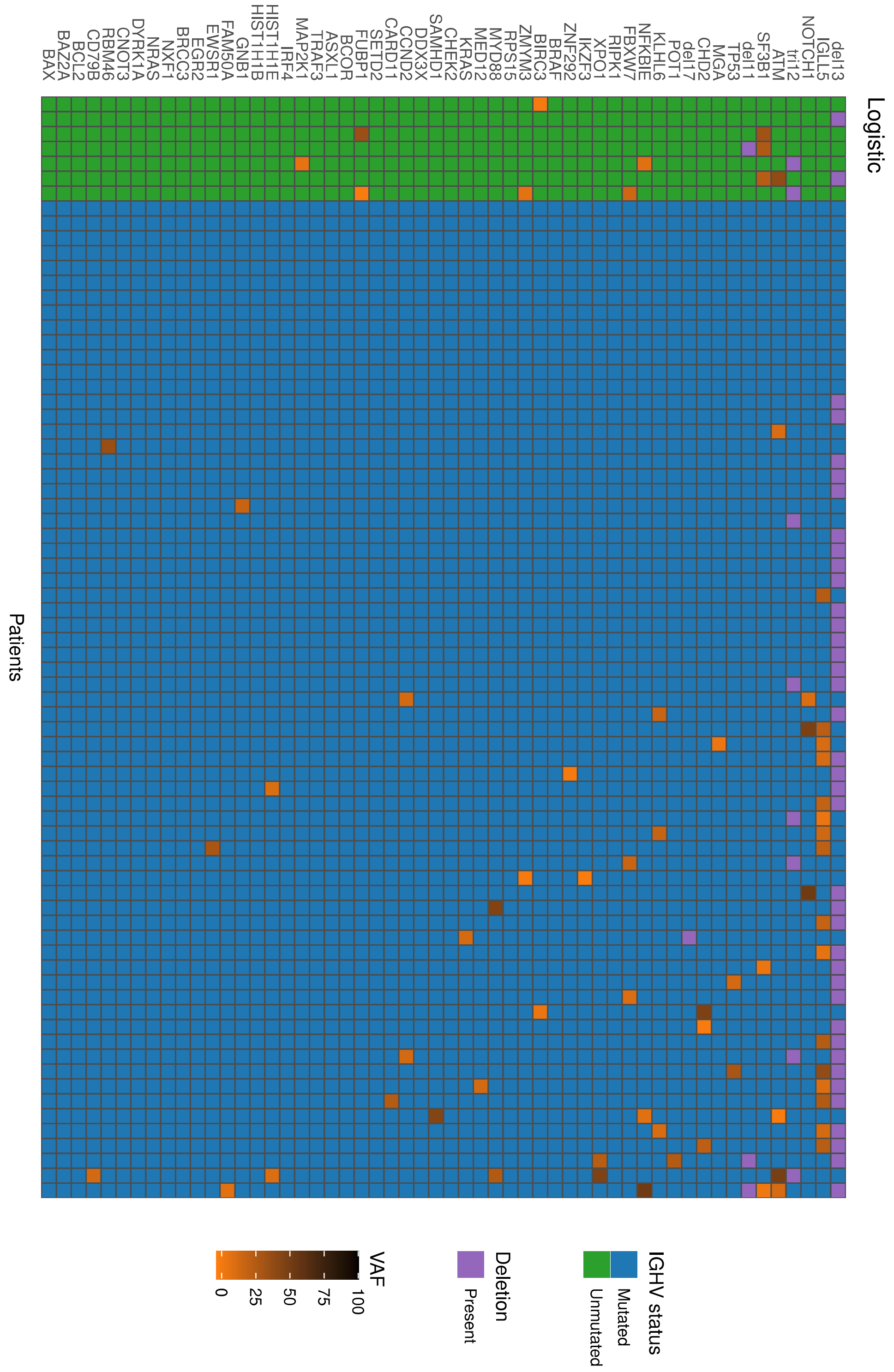

Supplementary Figure S7 *Growth dynamics and genetic changes in naturally progressing CLL.*

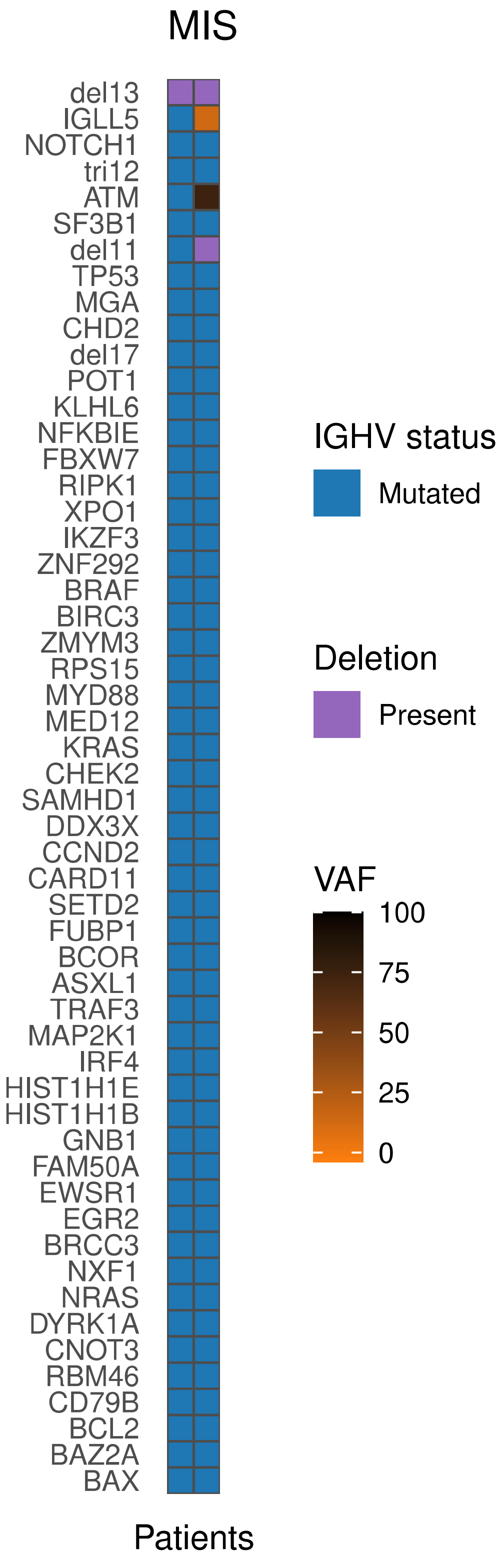

Supplementary Figure S8 *Growth dynamics and genetic changes in naturally progressing CLL.*

Variable	Total	Low slope group	Medium slope group	High slope group
Patients, n	1123	283	282	283
Age, median (IQR)	69 (62;77)	70 (65;77)	70 (62;75.75)	68 (61;78)
Sex, n (%)	668 (59 %)	172 (61 %)	163 (58 %)	158 (56 %)
Follow-up, median (IQR)	2.55 (1.17;4.69)	2.82 (1.4;4.81)	2.78 (1.42;4.78)	1.92 (0.78;3.49)
Follow-up before, median (IQR)	-2.92 (-8.13;-0.06)			
CLL-IPI 0-1, n (%)	635 (57 %)	179 (63 %)	178 (63 %)	133 (47 %)
2-3	281 (25 %)	51 (18 %)	60 (21 %)	93 (33 %)
4-10	131 (12 %)	21 (7 %)	30 (11 %)	41 (14 %)
Missing	76 (7 %)	32 (11 %)	14 (5 %)	16 (6 %)
ALC measurements before diagnosis, n [median (IQR)]	4073 [6.07 (3.18;10.9)]	1710 [5.29 (3.16;7.4)]	1157 [7.44 (3.54;11.36)]	1067 [14.4 (3.58;27.65)]
ALC measurements after diagnosis, n [median (IQR)]	16958 [6.44 (1.1;22.56)]	3540 [5.6 (1.54;10.59)]	3106 [13.6 (4.66;27.07)]	5128 [5.63 (0.81;32.9)]

Supplementary Table S1 Patient characteristics of the 1123 patients who were followed.

Model	Submodel	Covariate	Estimate	Type of estimate
Linear mixed model	Fixed effects	Intercept	2.59 (2.53-2.65)	Estimates (95% confidence intervals)
Linear mixed model	Fixed effects	Time	0.4 (0.36-0.43)	Estimates (95% confidence intervals)
Linear mixed model	Random effects	Intercept	0.81	Standard deviation
Linear mixed model	Random effects	Time	0.27	Standard deviation
Linear mixed model	Random effects	Time	0.73	Correlation
Competing risk models	Death	Ipi	1.29 (1.17-1.42)	HR (95% confidence intervals)
Competing risk models	Death	Gender male	1.06 (0.71-1.58)	HR (95% confidence intervals)
Competing risk models	Death	High slope group	1.64 (0.97-2.76)	HR (95% confidence intervals)
Competing risk models	Death	Medium slope group	1.45 (0.87-2.4)	HR (95% confidence intervals)
Competing risk models	Treatment	Ipi	1.32 (1.23-1.41)	HR (95% confidence intervals)
Competing risk models	Treatment	Gender male	1.27 (0.94-1.71)	HR (95% confidence intervals)
Competing risk models	Treatment	High slope group	2.45 (1.69-3.54)	HR (95% confidence intervals)
Competing risk models	Treatment	Medium slope group	0.94 (0.61-1.44)	HR (95% confidence intervals)

Supplementary Table S2 *Model specifications of the included models in the paper.*
