# Supplementary material for: Pre-diagnostic trajectories of lymphocytosis predict time to treatment and death in patients with chronic lymphocytic leukemia
Source: Commun Med (Lond). 2022 May 12;2:50. doi: 10.1038/s43856-022-00117-4 (PMC9098503; doi:10.1038/s43856-022-00117-4)
Supplement: Supplementary file 3 — Reporting Summary [file 43856_2022_117_MOESM3_ESM.pdf]

nature research

Corresponding author(s): Carsten Utoft Niemann

Last updated by author(s): Apr 21, 2022

Reporting Summary

Nature Research wishes to improve the reproducibility of the work that we publish. This form provides structure for consistency and transparency in reporting. For further information on Nature Research policies, see our Editorial Policies and the Editorial Policy Checklist.

Statistics

For all statistical analyses, confirm that the following items are present in the figure legend, table legend, main text, or Methods section.

n/a	Confirmed	
☐	☒	The exact sample size (*n*) for each experimental group/condition, given as a discrete number and unit of measurement
☐	☒	A statement on whether measurements were taken from distinct samples or whether the same sample was measured repeatedly
☐	☒	The statistical test(s) used AND whether they are one- or two-sided<br>*Only common tests should be described solely by name; describe more complex techniques in the Methods section.*
☐	☒	A description of all covariates tested
☐	☒	A description of any assumptions or corrections, such as tests of normality and adjustment for multiple comparisons
☐	☒	A full description of the statistical parameters including central tendency (e.g. means) or other basic estimates (e.g. regression coefficient) AND variation (e.g. standard deviation) or associated estimates of uncertainty (e.g. confidence intervals)
☒	☐	For null hypothesis testing, the test statistic (e.g. *F*, *t*, *r*) with confidence intervals, effect sizes, degrees of freedom and *P* value noted<br>*Give P values as exact values whenever suitable.*
☒	☐	For Bayesian analysis, information on the choice of priors and Markov chain Monte Carlo settings
☒	☐	For hierarchical and complex designs, identification of the appropriate level for tests and full reporting of outcomes
☒	☐	Estimates of effect sizes (e.g. Cohen's *d*, Pearson's *r*), indicating how they were calculated

Our web collection on statistics for biologists contains articles on many of the points above.

Software and code

Policy information about availability of computer code

Data collection	NA
Data analysis	R version 4.0 was used for the data analysis of this study.

For manuscripts utilizing custom algorithms or software that are central to the research but not yet described in published literature, software must be made available to editors and reviewers. We strongly encourage code deposition in a community repository (e.g. GitHub). See the Nature Research guidelines for submitting code & software for further information.

Data

Policy information about availability of data

All manuscripts must include a data availability statement. This statement should provide the following information, where applicable:

- Accession codes, unique identifiers, or web links for publicly available datasets
- A list of figures that have associated raw data
- A description of any restrictions on data availability

Data availability
Restrictions apply to the availability of the underlying data sets, which were used under specific conditions for the current study. The reason for not making the data set publicly available is that danish law restrict publication of any individual level data and group with less than five persons. Moreover, according to Danish legislation, sequencing data cannot be fully anonymized, and data cannot be shared in a repository. All danish registry data can be obtained through application to the relevant data agencies and sequencing data can be obtained by contacting Christian.brighel@regionh.dk [18].
Code availability
Analytical methods and source code are stored https://github.com/masger/cllprognosis. The app is available at https://clllab.shinyapps.io/cllprognosis/. The source

code (DOI: 10.5281/zenodo.6470207) for the app is available at: https://github.com/masger/cllprognosis [33].

Field-specific reporting

Please select the one below that is the best fit for your research. If you are not sure, read the appropriate sections before making your selection.

☒ Life sciences ☐ Behavioural & social sciences ☐ Ecological, evolutionary & environmental sciences

For a reference copy of the document with all sections, see nature.com/documents/nr-reporting-summary-flat.pdf

Life sciences study design

All studies must disclose on these points even when the disclosure is negative.

Sample size	Retrospective CLL cohort: We included all patients diagnosed with CLL in Greater Copenhagen between 2008 and 2016 through the Danish CLL Register (DCLLR).
Data exclusions	We excluded 248 patients who did not have at least one observation within five years of diagnosis. Low slope groups had a growth rate of < 0.32 x 109 lymphocytes/ L / per year, compared with the high slope group with a growth rate of > 0.44 x 109 lymphocytes / L / per year.
Replication	NA
Randomization	NA
Blinding	NA

Reporting for specific materials, systems and methods

We require information from authors about some types of materials, experimental systems and methods used in many studies. Here, indicate whether each material, system or method listed is relevant to your study. If you are not sure if a list item applies to your research, read the appropriate section before selecting a response.

Materials & experimental systems

n/a	Involved in the study	
☒	☐	Antibodies
☒	☐	Eukaryotic cell lines
☒	☐	Palaeontology and archaeology
☒	☐	Animals and other organisms
☒	☐	Human research participants
☐	☒	Clinical data
☒	☐	Dual use research of concern

Methods

n/a	Involved in the study	
☒	☐	ChIP-seq
☒	☐	Flow cytometry
☒	☐	MRI-based neuroimaging

Clinical data

Policy information about clinical studies

All manuscripts should comply with the ICMJE guidelines for publication of clinical research and a completed CONSORT checklist must be included with all submissions.

Clinical trial registration	NA
Study protocol	NA
Data collection	Retrospective CLL cohort: We included all patients diagnosed with CLL in Greater Copenhagen between 2008 and 2016 through the Danish CLL Register (DCLLR). Since 2008, all newly diagnosed CLL patients are registered in the DCLLR [23]. All hematological departments in Denmark are required to report clinical data for patients with CLL and the data completeness in the DCLLR is 98.3% [23]. The DCLLR holds information on birthday, treating hospital, prognostic markers and treatment initiations. From this database, we obtained information regarding age at diagnosis, Binet stage, β-2-microglobulin, fluorescence in situ hybridization data, IGHV mutational status and date of first line treatment or death (if applicable). CLL-IPI was calculated using B2M cutoff of 4 and del(17p) as the only TP53 aberration [8].
Outcomes	The timescale was time from diagnosis until the first event of death or treatment.
